# Supplementary material for: A Strategy for Selecting “Q-Markers” of Chinese Medical Preparation via Components Transfer Process Analysis with Application to the Quality Control of Shengmai Injection
Source: Molecules. 2019 May 10;24(9):1811. doi: 10.3390/molecules24091811 (PMC6539032; doi:10.3390/molecules24091811)
Supplement: Supplementary file 1 [file molecules-24-01811-s001.zip › supplementary files/supporting date.docx]

*Article*

**A strategy for selecting “Q-markers” of Chinese medical preparation via components transfer process analysis with application to the quality control of Shengmai injection**

**Chunxia Zhao^1,#^, Huan Liu ^1,#^, Peiqi Miao ^1^, Houen Wang ^1^, Heshui Yu ^1,2^, Chunhua Wang ^1,2,*^, Zheng Li ^1,2,*^**

^1^College of Pharmaceutical Engineering of Traditional Chinese Medicine, Tianjin University of Traditional Chinese Medicine Tianjin 301617, China

zhaochunxia199411@163.com (C.Z.); [13512039643@163.com](mailto:13512039643@163.com) (H.L.)

[miaopeiqi@163.com(P.M.)](mailto:miaopeiqi@163.com(P.M.)); [13072200650@126.com](mailto:13072200650@126.com) (H.W)

hs_yu08@163.com (H.Y.);

^2^Tianjin Key Laboratory of Modern Chinese Medicine, Tianjin University of Traditional Chinese Medicine, Tianjin 301617, China

#These authors contributed equally to this work

*Corresponding authors. Email: [pharmwch@126.com](mailto:pharmwch@126.com) (C. Wang) and lizheng@tjutcm.edu.cn (Z. Li). Phone/Fax: +86-22-2738-6453

TableS 1- the identified chemical constituents of SMI

| **No.** | Rt (min) | Name | Formula | Theoretical molecular weight | Actual molecular weight | Error (ppm) | Pseudo  molecular ion (+) | Pseudo  molecular ion (-) | Fragments (+) | Fragments (-) | Plant materia |
| --- | --- | --- | --- | --- | --- | --- | --- | --- | --- | --- | --- |
| **1** | 1.105 | 5-Hydroxymethyl  furfural | C_6_H_6_O_3_ | 126.11  100 |  |  | 127.0403  [M+H]^+^ |  | 109.0296 |  | FS |
| **2** | 1.753 | 3-Hydroxymethyl-2-furfural | C_6_H_6_O_3_ | 126.1100 |  |  | 127.0404  [M+H]^+^  144.0817  [M+NH4]^+^ |  | 109.0303  111.0456 |  | FS |
| **3** | 4.579 | Oleanolic acid | C_30_H_48_O_3_ | 456.7003 |  |  | 457.3683  [M+H]^+^ |  | 439.3578  421.3484 |  | RG |
| **4** | 6.165 | Neokadsuranic acid B | C_33_H_44_O_3_ | 488.7007 |  |  | 453.3445  [M+H]^+^  475.3260  [M+Na]^+^ |  | 435.3328  339.2723  227.1785 |  | FS |
| **5** | 6.311 | Majonoside R1/Momorcharassikde A | C_42_H_72_O_15_ | 817.0121 | 815.4793 | -2.3 |  | 861.4831  [M+COOH]^-^  815.4774  [M-H]^-^ |  | 653.2431  553.3397  521.2028  391.1734 | RG |
| **6** | 6.567 | Notoginsenoside N/M/R6/20-glc-ginsenoside Rf | C_48_H_82_O_19_ | 963.1533 | 961.5372 | -1.4 |  | 1007.5421  [M+COOH]^-^  961.5358  [M-H]^-^ |  | 799.4791  781.4736  637.4299  619.2382  475.1818 | RG |
| **7** | 6.567 | Quinquenoside F1 | C_42_H_74_O_15_ | 819.0280 | 817.4949 | -1.6 |  | 863.4997  [M+COOH]^-^  817.4937  [M-H]^-^ |  | 655.4421  493.3854 | RG |
| **8** | 7.317 | Citral | C_10_H_16_O | 152.2334 |  |  | 153.1297  [M+H]^+^ |  | 107.0874 |  | FS |
| **9** | 8.227 | Notoginsenoside Ft1 | C_47_H_80_O_17_ | 917.1279 |  | -3.3 |  | 961.5340  [M+COOH]^-^ |  |  | RG |
| **10** | 8.366 | Quinquenoside F1 | C_42_H_74_O_15_ | 819.0280 | 817.4949 | -1.2 |  | 863.4985  [M+COOH]^-^  817.4939  [M-H]^-^ |  | 655.4413  493.1465 | RG |
| **11** | 8.522 | Gomisin M1/M2/L1/L2 | C_22_H_26_O_6_ | 386.4382 |  |  | 387.1805  [M+H]^+^ |  | 372.1577  355.1549  345.1341  331.1198  299.0917 |  | FS |
| **12** | 8.583 | Majonoside R1/Momorcharassikde A | C_42_H_72_O_15_ | 817.0121 | 815.4793 | 1.8 |  | 861.4769  [M+COOH]^-^  815.4808  [M-H]^-^ |  | 653.4280  553.3405  391.2906 | RG |
| **13**  **14** | 9.404  9.404 | Notoginsenoside R1  Ginsenoside Rha | C_47_H_80_O_18_  C_53_H_90_O_22_ | 933.1273  1079.2685 | 931.5266  1077.5845 | -0.3  -0.8 |  | 977.5306 [M+COOH]^-^  931.5263  [M-H]^-^  1123.5892  [M+COOH]^-^  1077.5836  [M-H]^-^ |  | 799.4839  769.2535  751.3068  637.4327  475.3794 | RG |
| **15** | 9.518 | Notoginsenoside N/M/R6/20-glc-Rf | C_48_H_82_O_19_ | 963.1533 | 961.5372 | 0.4 |  | 1007.5819  [M+COOH]^-^  961.5376  [M-H]^-^ |  | 799.4842  781.4769  637.4319  619.4381  475.3811 | RG |
| **16** | 9.678 | Notoginsenoside R1 | C_47_H_80_O_18_ | 933.1273 | 931.5266 | -0.2 |  | 977.5303 [M+COOH]^-^  931.5264  [M-H]^-^ |  | 799.4824  769.4754  751.2925  637.4317  475.3773 | RG |
| **17** | 9.751 | Notoginsenoside N/M/R6/20-glc-Rf | C_48_H_82_O_19_ | 963.1533 | 961.5372 | -0.9 |  | 961.5363  [M-H]^-^ |  | 799.4834  637.4253  475.3763 | RG |
| **18** | 9.853 | Notoginsenoside R1 | C_47_H_80_O_18_ | 933.1273 | 931.5266 | -0.6 |  | 977.5323 [M+COOH]^-^  931.5269  [M-H]^-^ |  | 799.4839  769.4730  751.4630  637.4307  475.2178 | RG |
| **19** | 10.002 | Notoginsenoside N/M/R6/20-glc-Rf | C_48_H_82_O_19_ | 963.1533 | 961.5372 | -2.7 |  | 1007.5428  [M+COOH]^-^  961.5346  [M-H]^-^ |  | 799.4824  781.4721  637.4290  619.4393  475.3716 | RG |
| **20** | 10.126 | Notoginsenoside R1 | C_47_H_80_O_18_ | 933.1273 | 931.5266 | -4.1 |  | 977.5260 [M+COOH]^-^  931.5228  [M-H]^-^ |  | 799.4812  769.4669  751.2809  637.4295  475.0670 | RG |
| **21** | 10.536 | Ginsenoside Re | C_48_H_82_O_18_ | 947.1539 | 945.5423 | -1.4 |  | 991.5456 [M+COOH]^-^ 945.5410  [M-H]^-^ |  | 799.4835  783.4885  765.4759  637.4313  619.4208  475.3783 | RG |
| **22** | 10.536 | Ginsenoside Rg1 | C_42_H_72_O_14_ | 801.0127 | 799.4844 | -1.1 |  | 845.4885 [M+COOH]^-^  799.4835  [M-H]^-^ |  | 637.4313  619.4207  475.3784 | RG |
| **23** | 10.649 | L-borneol-7-O-[β-D-apiofuranosyl(1→6)]- β-D-glucopyranoside | C_21_H_36_O_10_ | 448.5045 | 447.2230 | 0.4 |  | 493.2288  [M+COOH]^-^ 447.2232  [M-H]^-^ |  | 315.1813 | RO |
| **24** | 12.278 | Notoginsenoside R2/F3/F5 | C_41_H_70_O_23_ | 930.9807 | 769.4738 | -1.7 |  | 815.4780 [M+COOH]^-^  769.4725  [M-H]^-^ |  | 637.4309  619.4201  475.3795 | RG |
| **25** | 12.278 | Ace-ginsenoside Rg1 | C_44_H_74_O_15_ | 843.0494 | 841.4949 | -1.7 |  | 887.4987  [M+COOH]^-^  841.4935  [M-H]^-^ |  | 841.4935  799.4832  781.4727  637.4310  619.4202  475.3791 | RG |
| **26** | 12.466 | Ace-ginsenoside Rf | C_44_H_74_O_15_ | 843.0494 | 841.4949 | -1.8 |  | 887.4986  [M+COOH]^-^ 841.4934  [M-H]^-^ |  | 841.4933  799.4829  781.4726  637.3069  619.4210  475.3783 | RG |
| **27** | 12.470 | Notoginsenoside N/M/R6/20-glc-Rf | C_48_H_82_O_19_ | 963.1533 | 961.5372 | -1.8 |  | 1007.5388  [M+COOH]^-^  961.5355  [M-H]^-^ |  | 799.4838  781.4721  637.4313  619.4186  475.3783 | RG |
| **28** | 12.913 | Nigranoic acid | C_30_H_46_O_4_ | 470.6838 |  |  | 471.3459  [M+H]^+^ |  | 133.1021  145.0508  159.1171  173.1330 |  | FS |
| **29** | 12.936 | Notoginsenoside N/M/R6/20-glc-Rf | C_48_H_82_O_19_ | 963.1533 | 961.5372 | -2.8 |  | 1007.5383  [M+COOH]^-^  961.5347  [M-H]^-^ |  | 799.4794  781.4698  637.4270  619.4228  475.3733 | RG |
| **30** | 12.986 | Notoginsenoside R2/F3/F5 | C_41_H_70_O_23_ | 930.9807 |  |  |  | 815.4778 [M+COOH]^-^  769.4744  [M-H]^-^ |  | 637.4280  619.4239  475.1832 | RG |
| **31** | 13.082 | Quinquenoside III | C_51_H_86_O_21_ | 1035.2159 | 987.5529 | -1.9 |  | 1033.5549  [M+COOH]^-^  987.5510  [M-H]^-^ |  | 945.5407  783.4875  765.4809  621.2800  459.1711 | RG |
| **32** | 13.388 | Ginsenoside Rg2 | C_42_H_72_O_13_ | 785.0133 |  |  |  | 829.4936 [M+COOH]^-^ 783.4858  [M-H]^-^ |  | 637.4295  619.4204  475.3777 | RG |
| **33** | 13.703 | Glucosinic acid | C_30_H_46_O_3_ | 454.6844 |  |  | 455.3528  [M+H]^+^ |  | 437.3410  187.1495  175.1491  161.1341 |  | FS |
| **34** | 13.950 | Ginsenoside Rf | C_42_H_72_O_14_ | 801.0127 | 799.4844 | -1.4 |  | 845.4881  [M+COOH]^-^  799.4833  [M-H]^-^ |  | 637.4312  619.4200  475.3786 | RG |
| **35** | 14.157 | Notoginsenoside R4/Ra3/Fa | C_59_H_100_O_27_ | 1241.4091 | 1239.6374 | -1.0 |  | 1285.6155  [M+COOH]^-^ 1239.6361  [M-H]^-^ |  | 1107.5936  1077.5874  945.5415  783.4885  765.4800  621.4368  459.3844 | RG |
| **36** | 14.392 | Ginsenoside Rf | C_42_H_72_O_14_ | 801.0127 | 799.4844 | 0.0 |  | 845.4896  [M+COOH]^-^  799.4844  [M-H]^-^ |  | 637.4323  619.3134  475.3782 | RG |
| **37** | 14.392 | Notoginsenoside Q | C_63_H_106_O_30_ | 1343.4977 | 1341.6597 | -1.5 |  | 1341.6648  [M-H]^-^ |  | 1209.6302  1077.5874  1047.5726  945.5390  783.4884  765.4772  621.4347  459.3809 | RG |
| **38** | 14.797 | Notoginsenoside R2/F3/F5 | C_41_H_70_O_13_ | 770.9867 | 769.4738 | -0.6 |  | 815.4790 [M+COOH]^-^  769.4733  [M-H]^-^ |  | 637.4318  619.4218  475.3790 | RG |
| **39** | 14.844 | Notoginsenoside R4/Ra3/Fa | C_59_H_100_O_27_ | 1241.4091 | 1239.6374 | -3.5 |  | 1239.6331  [M-H]^-^ |  | 1107.5883  1077.5829  945.5391  783.4873  765.4896  621.4347  459.3842 | RG |
| **40** | 15.573 | Quadrangulcoside | C_54_H_90_O_23_ | 1107.2786 | 1105.5795 | -1.7 |  | 1105.5775  [M-H]^-^ |  | 943.5251  781.4737  763.4636  619.4199  457.3692 | RG |
| **41** | 15.972 | Notoginsenoside Fc/Ra1/Ra2 | C_58_H_98_O_26_ | 1211.3831 | 1209.6209 | 0.2 |  | 1209.6211  [M-H]^-^ |  | 1077.5820  1047.5768  945.5323  915.5342  783.4874  765.4772  621.4296  603.4311  459.3806 | RG |
| **42** | 15.972 | Notoginsenoside R4/Ra3/Fa | C_59_H_100_O_27_ | 1241.4091 | 1239.6374 | -3.5 |  | 1239.6331  [M-H]^-^ |  | 1107.5905  1077.5864  945.5323  783.4875  765.4777  621.4280  459.3806 | RG |
| **43** | 15.972 | Ginsenoside Rg2 | C_42_H_72_O_13_ | 785.0133 | 783.4895 | -2.3 |  | 829.4931 [M+COOH]^-^ 783.4877  [M-H]^-^ |  | 637.4306  619.4200  475.3785 | RG |
| **44** | 15.972 | Ginsenoside Rh1 | C_36_H_62_O_9_ | 638.8721 | 637.4316 | -1.6 | + | 683.4366 [M+COOH]^-^  637.4306  [M-H]^-^  1275.8663  [2M-H]^-^ |  | 475.3784 | RG |
| **45** | 16.303 | Notoginsenoside Fc/Ra1/Ra2 | C_58_H_98_O_26_ | 1211.3831 | 1209.6268 | -1.3 |  | 1209.6252  [M-H]^-^ |  | 1077.5806  1047.5712  945.5413  915.5315  783.4888  765.4784  621.4366  603.4294  459.3836 | RG |
| **46** | 16.744 | Ginsenoside Rg2/F2 | C_42_H_72_O_13_ | 785.0133 | 783.4895 | -0.9 |  | 829.4944 [M+COOH]^-^ 783.4888  [M-H]^-^ |  | 637.4316  619.4213  475.3791 | RG |
| **47** | 16.744 | Ginsenoside Rb1 isomer | C_54_H_92_O_23_ | 1109.2945 | 1107.5951 | -2.4 |  | 1153.5930  [M+COOH]^-^  1107.5924  [M-H]^-^ |  | 945.5407  783.4893  765.4786  621.4365  603.4312  459.3843 | RG |
| **48** | 16.744 | Notoginsenoside R4/Ra3/Fa | C_59_H_100_O_27_ | 1241.4091 | 1239.6374 | -2.5 |  | 1275.8663  [M+COOH]^-^  1239.6343  [M-H]^-^ |  | 1107.5931  1077.5829  945.5406  783.4889  765.4795  621.4363  459.3839 | RG |
| **49** | 17.026 | Ginsenoside Rb1 | C_54_H_92_O_23_ | 1109.2945 | 1107.5951 | -2.5 |  | 1153.5925  [M+COOH]^-^  1107.5923  [M-H]^-^ |  | 945.5410  783.4879  765.4752  621.4349  603.4188  459.3814 | RG |
| **50** | 17.037 | Mal-ginsenoside Rd | C_51_H_84_O_21_ | 1033.2001 | 1031.5427 | 2.1 |  | 1077.5782  [M+COOH]^-^  1031.5449  [M-H]^-^ |  | 945.5397  783.4885  765.4738  621.4348  459.3800 | RG |
| **51** | 17.051 | Ginsenoside F1/Rh1 | C_36_H_62_O_9_ | 638.8721 | 637.4316 | -0.6 |  | 683.4365 [M+COOH]^-^  637.4312  [M-H]^-^  1275.8662  [2M-H]^-^ |  | 475.3784 | RG |
| **52** | 17.528 | Mal-ginsenoside Rb1 | C_57_H_94_O_26_ | 1195.3407 | 1193.5955 | -1.6 |  | 1239.6329  [M+COOH]^-^  1193.5938  [M-H]^-^ |  | 1149.6061  1107.5950  1089.5836  945.5453  927.5250  783.4896  765.4796  621.4360  459.3821 | RG |
| **53** | 17.748 | Ace-ginsenoside Rb1/Quinquenoside R1 | C_56_H_94_O_24_ | 1151.3312 | 1149.6057 | -1.0 |  | 1195.6368  [M+COOH]^-^  1149.6046  [M-H]^-^ |  | 1107.5947  1089.5837  945.5456  927.5250  783.4906  765.4791  621.4381  459.3821 | RG |
| **54** | 17.748 | Tuberoside A/Amaramthussaponin | C_48_H_76_O_19_ | 957.1056 | 955.4903 | 0.5 |  | 955.4908  [M-H]^-^ |  | 731.4353  613.3730  587.4069  569.3745  523.3809  455.3567 | RG |
| **55** | 17.926 | Ginsenoside Ro | C_48_H_76_O_19_ | 957.1056 | 955.4903 | -1.3 |  | 955.4891  [M-H]^-^ |  | 793.4346  631.2678  455.1756 | RG |
| **56** | 18.584 | Ginsenoside Rc | C_53_H_90_O_22_ | 1079.2685 | 1107.5951 | -2.4 |  | 1123.5824  [M+COOH]^-^  1077.5830  [M-H]^-^ |  | 945.5408  915.5286  783.4886  621.4356  459.3879 | RG |
| **57** | 18.688 | Notoginsenoside Fc/Ra1/Ra2 | C_58_H_98_O_26_ | 1211.3831 | 1209.6268 | -1.1 |  | 1255.5912  [M+COOH]^-^  1209.6252  [M-H]^-^ |  | 1077.5836  1047.5646  945.5413  915.5310  783.4894  765.4786  621.4372  603.4270  459.3852 | RG |
| **58** | 19.453 | Mal-ginsenoside Rb2/Rb3/Rc | C_56_H_92_O_25_ | 1165.3147 | 1163.5849 | -1.5 |  | 1209.6266  [M+COOH]^-^ 1163.5831  [M-H]^-^ |  | 1119.5962  1077.5854  1059.5748  945.5447  915.5191  783.4868  765.4830  621.4327  459.3786 | RG |
| **59** | 19.994 | Notoginsenoside Fc/Ra1/Ra2 | C_58_H_98_O_26_ | 1211.3831 | 1209.6268 | 0.2 |  | 1255.6200  [M+COOH]^-^ 1209.6270  [M-H]^-^ |  | 1077.5841  1047.5728  945.5438  915.5299  783.4911  765.4810  621.4388  603.4271  459.3857 | RG |
| **60** | 19.994 | Ace-ginsenoside Rb1/Quinquenoside R1 | C_56_H_94_O_24_ | 1151.3312 | 1149.6057 | 0.4 |  | 1149.6062  [M-H]^-^ |  | 1107.5964  1089.5869  945.5428  927.5196  783.4907  765.4789  621.4390  459.3847 | RG |
| **61** | 20.289 | Stipuleanoside R1 | C_47_H_74_O_18_ | 927.0797 | 925.4797 | -0.5 |  | 971.5169  [M+COOH]^-^ 925.4792  [M-H]^-^ |  | 763.4269  613.3699  587.4039  569.3914  455.3524 | RG |
| **62** | 20.492 | Chikusetsu saponin IV | C_47_H_74_O_18_ | 927.0797 | 925.0797 | -0.5 |  | 971.5169  [M+COOH]^-^ 925.4792  [M-H]^-^ |  | 775.4359  613.3746  569.4030  492.1461 | RG |
| **63** | 20.791 | Notoginsenoside Fc/Ra1/Ra2 | C_58_H_98_O_26_ | 1211.3831 |  |  |  | 1209.6246  [M-H]^-^ |  | 1077.5831  1047.5780  945.5403  915.5320  783.4879  765.4760  621.4370  603.4268  459.3841 | RG |
| **64** | 20.926 | Ginsenoside Rb2 | C_53_H_90_O_22_ | 1079.2685 | 1077.5845 | -1.8 |  | 1123.5868  [M+COOH]^-^ 1077.5826  [M-H]^-^ |  | 945.5396  915.5304  783.4889  621.4359  459.3828 | RG |
| **65** | 20.984 | Chikusetsu saponin IV | C_47_H_74_O_18_ | 927.0797 | 925.4797 | -0.3 |  | 971.4319  [M+COOH]^-^ 925.4782  [M-H]^-^ |  | 775.4300  613.3729  569.3840  492.1411 | RG |
| **66** | 21.595 | Ginsenoside Rb3 | C_53_H_90_O_22_ | 1079.2685 | 1077.5845 | -0.7 |  | 1123.5837  [M+COOH]^-^ 1077.5837  [M-H]^-^ |  | 945.5394  915.5297  783.4905  621.4379 | RG |
| **67** | 21.852 | Mal-ginsenoside Rb2/Rb3/Rc | C_56_H_92_O_25_ | 1165.3147 | 1163.5791 | 0.6 |  | 1209.5885  [M+COOH]^-^ 1163.5797  [M-H]^-^ |  | 1119.5930  1077.5825  1059.5712  945.5435  915.5320  783.4827  765.4830  621.4379  459.3854 | RG |
| **68** | 21.910 | Ginsenoside Rs1/Rs2 | C_55_H_92_O_23_ | 1121.3052 | 1119.5951 | -1.9 |  | 1119.5935  [M-H]^-^ |  | 1077.5822  1059.5746  945.5361  915.5380  783.4869  765.4836 | RG |
| **69** | 22.432 | Notoginsenoside Fc/Ra1/Ra2 | C_58_H_98_O_26_ | 1211.3831 | 1209.6268 | -2.0 |  | 1255.5917  [M+COOH]^-^ 1209.6244  [M-H]^-^ |  | 1077.5824  1047.5701  945.5349  915.5283  783.4885  765.4788  621.4373  603.4232  459.3866 | RG |
| **70** | 22.432 | Ginsenoside Rs1/Rs2 | C_55_H_92_O_23_ | 1121.3052 | 1119.5951 | -2.0 |  | 1165.5674  [M+COOH]^-^  1119.5927  [M-H]^-^ |  | 1077.5823  1059.5712  945.5346  915.5278  783.4886  765.4791  621.4374  459.3866 | RG |
| **71** | 22.683 | Schisandrin/  Gomisin S/Isoschizandrin/Schizandrol A | C_24_H_32_O_7_ | 432.5067 |  |  | 433.2212  [M+H]^+^ |  | 415.2128  384.1946 |  | FS |
| **72** | 22.766 | Cynarasaponin C/Spinasaponin A | C_35_H_70_O_19_ | 794.9189 | 793.4374 | -1.4 |  | 793.4363  [M-H]^-^ |  | 673.3953  631.3841  613.3727  587.3909  569.3853  497.3553  455.3521 | RG |
| **73** | 22.766 | Ace-ginsenoside Ra1/Ra2 | C_60_H_100_O_27_ | 1253.4198 | 1251.6374 | -2.6 |  | 1251.6343  [M-H]^-^ |  | 1209.6238  1077.5815  1059.5759  945.5365  915.3580  783.4876  765.4770  621.4375  459.3867 | RG |
| **74** | 22.766 | Chikusetsu saponin IVa | C_42_H_66_O_14_ | 794.9650 | 793.4374 | -1.4 |  | 793.4363  [M-H]^-^ |  | 631.3840 | RG |
| **75** | 22.766 | Ginsenoside Rs1/Rs2 | C_55_H_92_O_23_ | 1121.3052 | 1119.5951 | -3.8 |  | 1119.5909  [M-H]^-^ |  | 1077.5801  1059.5690  945.5386  915.5223  783.4877  765.4776  621.4345  459.3855 | RG |
| **76** | 22.848 | Schisandrin/  Gomisin S/Isoschizandrin/Schizandrol A | C_24_H_32_O_7_ | 432.5067 |  |  | 455.2054  [M+Na]^+^ |  | 415.2139  384.1940  369.1709  346.1420  353.1750  216.1853 |  | FS |
| **77** | 23.746 | Ginsenoside Rs1/Rs2 | C_55_H_92_O_23_ | 1121.3052 | 1119.5951 | 0.0 |  | 1119.5917  [M-H]^-^ |  | 1077.5817  1059.5674  945.5389  915.3846  783.4864  765.4250  621.4348 | RG |
| **78** | 24.336 | Ginsenoside Rd | C_48_H_82_O_18_ | 947.1539 | 945.5423 | -0.1 |  | 991.5475  [M+COOH]^-^ 945.5422  [M-H]^-^ |  | 783.4891  765.4796  621.4373  603.4274  459.3840 | RG |
| **79** | 24.336 | Ace-ginsenoside Ra1/Ra2 | C_60_H_100_O_27_ | 1253.4198 | 1251.6374 | -1.8 |  | 1251.6348  [M-H]^-^ |  | 1209.6216  1077.5824  1059.5699  945.5415  915.5283  783.4883  765.4794  621.4362  459.3834 | RG |
| **80** | 24.372 | Schisandrin/  Gomisin S/Isoschizandrin/Schizandrol A | C_24_H_32_O_7_ | 432.5067 |  |  | 433.2038  [M+H]^+^ |  | 415.2020  384.1913 |  | FS |
| **81** | 26.176 | Epigomisin O | C_23_H_28_O_7_ | 416.4642 |  |  | 439.1743  [M+Na]^+^  417.1851  [M+H]^+^ |  | 399.1792  330.1110  353.1393  315.0870 |  | FS |
| **82** | 26.180 | Ginsenoside Rd isomer | C_48_H_82_O_18_ | 947.1539 | 945.5423 | -2.0 |  | 991.5458 [M+COOH]^-^ 945.5404  [M-H]^-^ |  | 783.4869  765.4753  621.4350  603.4334  459.3888 | RG |
| **83** | 26.189 | Schisandrin/  Gomisin S/Isoschizandrin/Schizandrol A | C_24_H_32_O_7_ | 432.5067 |  |  | 433.1977  [M+H]^+^ |  | 415.1927  384.1588 |  | FS |
| **84** | 26.408 | Ginsenoside Rg8/Rg9 | C_42_H_70_O_13_ | 782.9974 | 781.4738 | -1.4 |  | 827.4792  [M+COOH]^-^  781.4727  [M-H]^-^ |  | 619.4190 | RG |
| **85** | 27.227 | Ginsenoside Rg8/Rg9 | C_42_H_70_O_13_ | 782.9974 | 781.4738 | -2.4 |  | 827.4732  [M+COOH]^-^  781.4719  [M-H]^-^ |  | 619.4207 | RG |
| **86** | 28.981 | Ginsenoside F4 | C_42_H_70_O_12_ | 766.9980 | 765.4789 | -0.1 |  | 765.4788  [M-H]^-^ |  | 619.4207  601.4118  457.3639 | RG |
| **87** | 30.096 | Ginsenoside Rh4/Rk3 | C_36_H_60_O_8_ | 620.8568 | 619.4210 | 0.0 |  | 665.4258  [M+COOH]^-^  619.4210  [M-H]^-^ |  | 457.3700 | RG |
| **88** | 30.106 | Ginsenoside Rg6 | C_42_H_70_O_12_ | 766.9980 | 765.4789 | -0.8 |  | 765.4783  [M-H]^-^ |  | 619.4215  601.4114  457.3728 | RG |
| **89** | 31.043 | Ginsenoside Rh4/Rk3 | C_36_H_60_O_8_ | 620.8568 | 619.4210 | -0.2 |  | 665.4265  [M+COOH]^-^  619.4209  [M-H]^-^ |  | 457.3670 | RG |
| **90** | 31.413 | Chikusetsu saponin IVa | C_42_H_66_O_14_ | 794.9650 | 793.4374 | 1.6 |  | 793.4387  [M-H]^-^ |  | 731.4388  631.3867  613.3751  587.4146  569.3860  537.3511  523.3809  455.3523 | RG |
| **91** | 31.755 | Ginsenoside Rg3/Fc/F2 | C_42_H_72_O_13_ | 785.0133 | 783.4895 | -2.0 |  | 829.4942 [M+COOH]^-^ 783.4879  [M-H]^-^ |  | 621.4362  459.3850 | RG |
| **92** | 33.474 | Ginsenoside Rg3/Fc/F2 | C_42_H_72_O_13_ | 785.0133 | 783.4895 | -2.6 |  | 829.4922 [M+COOH]^-^ 783.4875  [M-H]^-^ |  | 621.4352  459.3831 | RG |
| **93** | 33.869 | Ginsenoside Rg3/Fc/F2 | C_42_H_72_O_13_ | 785.0133 | 783.4895 | -1.4 |  | 829.4933 [M+COOH]^-^ 783.4884  [M-H]^-^ |  | 621.4365  459.3834 | RG |
| **94** | 35.775 | Ginsenoside Rs3 | C_44_H_74_O_14_ | 827.0500 | 825.5000 | -2.2 |  | 871.5005  [M+COOH]^-^  825.4982  [M-H]^-^ |  | 783.4879  621.4351  459.3838 | RG |
| **95** | 36.160 | Ginsenoside Rs3 | C_44_H_74_O_14_ | 827.0500 | 825.5000 | -3.9 |  | 871.4998  [M+COOH]^-^  825.4968  [M-H]^-^ |  | 783.4866  621.4333  459.3851 | RG |
| **96** | 38.013 | g-Schizandrin | C_23_H_28_O_6_ | 400.4648 |  |  | 401.1971  [M+H]^+^ |  | 386.2129  370.2039  355.1917 |  | FS |
| **97** | 38.327 | Ginsenoside Rg5 | C_42_H_70_O_12_ | 766.9980 | 765.4789 | 0.5 |  | 811.4843  [M+COOH]^-^  765.4793  [M-H]^-^ |  | 603.4269  459.3750 | RG |
| **98** | 39.079 | Ginsenoside Rk1 | C_42_H_70_O_12_ | 766.9980 | 765.4789 | -1.0 |  | 811.4835  [M+COOH]^-^  765.4781  [M-H]^-^ |  | 603.4260  459.2032 | RG |
| **99** | 42.151 | Ginsenoside Rs4/ginsenoside Rs5/Ace-ginsenoside Rg5/Ace-ginsenoside Rk1 | C_44_H_72_O_13_ | 809.0347 | 807.4895 | 0.5 |  | 853.4960  [M+COOH]^-^  807.4899  [M-H]^-^ |  | 765.4799  747.4692  603.4272  537.3942  441.2397 | RG |
| **100** | 42.680 | Ginsenoside Rs4/ginsenoside Rs5/Ace-ginsenoside Rg5/Ace-ginsenoside Rk1 | C_44_H_72_O_13_ | 809.0347 | 807.4895 | 0.9 |  | 853.4969  [M+COOH]^-^  807.4902  [M-H]^-^ |  | 765.4800  747.4695  603.4281  537.3956  441.2296 | RG |

Note: FS: Fructus Schisandrae; RG: red ginseng; RO: radix ophiopogonis.

[1] Li F, Cheng T F, Dong X, et al. Global analysis of chemical constituents in Shengmai injection using high performance liquid chromatography coupled with tandem mass spectrometry[J].J Pharm Biomed Anal 2016;117:61-72.

[2] Wang Y H, Qiu C, Wang D W, et al. Identification of multiple constituents in the traditional Chinese medicine formula Sheng-Mai San and rat plasma after oral administration by HPLC–DAD–MS/MS[J].J Pharm Biomed Anal 2011;54(5):1110-1127.

[3] Wu F F, Sun H, Wei W F, et al. Rapid and global detection and characterization of the constituents in ShengMai San by ultra-performance liquid chromatography-high-definition mass spectrometry[J].J Sep Sci 2011;34(22):3194-3199.

[4] Wu L, Ding X P, Zhu D N, et al. Study on the radical scavengers in the traditional Chinese medicine formula shengmai san by HPLC-DAD coupled with chemiluminescence (CL) and ESI-MS/MS[J].J Pharm Biomed Anal 2010;52(4):438-445.

[5] Zheng C N, Hao H P, Wang X, et al. Diagnostic fragment-ion-based extension strategy for rapid screening and identification of serial components of homologous families contained in traditional Chinese medicine prescription using high-resolution LC-ESI-IT-TOF/MS: Shengmai injection as an example[J].J Mass Spectrom 2009;44(2):230-244.





Fig.S1- The identified Chemical structrues of ginsenosides in Shengmai injection.





Fig.S2- The identified chemical structures of lignans in Shengmai injection.





Fig.S3- The identified chemical structures of others in Shengmai injection.
